# Silver nanoparticles with plasma-polymerized hexamethyldisiloxane coating on 3D printed substrates are non-cytotoxic and effective against respiratory pathogens

**DOI:** 10.3389/fmicb.2023.1217617

**Published:** 2023-08-10

**Authors:** Anna Machková, Eva Vaňková, Klára Obrová, Paola Fürhacker, Tereza Košutová, Thomas Lion, Jan Hanuš, Vladimír Scholtz

**Affiliations:** ^1^Department of Physics and Measurements, Faculty of Chemical Engineering, University of Chemistry and Technology in Prague, Prague, Czechia; ^2^St. Anna Children’s Cancer Research Institute, Vienna, Austria; ^3^Department of Macromolecular Physics, Faculty of Mathematics and Physics, Charles University, Prague, Czechia; ^4^Department of Pediatrics, Medical University of Vienna, Vienna, Austria

**Keywords:** antimicrobial activity, antiviral activity, cold atmospheric plasma, magnetron sputtering, metallic coating, polylactic acid, *Pseudomonas aeruginosa*, rhinovirus

## Abstract

Due to the emerging resistance of microorganisms and viruses to conventional treatments, the importance of self-disinfecting materials is highly increasing. Such materials could be silver or its nanoparticles (AgNPs), both of which have been studied for their antimicrobial effect. In this study, we compared the biological effects of AgNP coatings with and without a plasma-polymerized hexamethyldisiloxane (ppHMDSO) protective film to smooth silver or copper coatings under three ambient conditions that mimic their potential medical use (dry or wet environments and an environment simulating the human body). The coatings were deposited on 3D printed polylactic acid substrates by DC magnetron sputtering, and their surface morphology was visualized using scanning electron microscopy. Cytotoxicity of the samples was evaluated using human lung epithelial cells A549. Furthermore, antibacterial activity was determined against the Gram-negative pathogenic bacterium *Pseudomonas aeruginosa* PAO1 and antiviral activity was assessed using human rhinovirus species A/type 2. The obtained results showed that overcoating of AgNPs with ppHMDSO creates the material with antibacterial and antiviral activity and at the same time without a cytotoxic effect for the surrounding tissue cells. These findings suggest that the production of 3D printed substrates coated with a layer of AgNPs-ppHMDSO could have potential applications in the medical field as functional materials.

## 1. Introduction

Unusual health threats emerging not only from the large immunological exclusion caused by the use of protective respiratory equipment during the COVID-19 (coronavirus disease 2019) pandemic but also the ever-increasing occurrence of bacterial resistance to antibiotics and virus mutations raise the need for new materials with antimicrobial properties. A very attractive option is the use of 3D printing for the inexpensive production of various environmentally friendly objects that possess excellent properties, especially in terms of strength and robustness.

The characteristics of 3D printed objects (i.e., adequate dimensional control, good surface finish and adaptability) make this technology interesting for various applications in fields such as healthcare, manufacturing, education, construction as well as home use (Tack et al., 2016; Roopavath and Kalaskar, 2017). This technology is based on high-temperature sintering of thermoplastic or polymer filaments and subsequent solidification of the printed product at room temperature. Polylactic acid (PLA) is one of the most commonly used polymers for 3D printing (Nagarajan et al., 2016). It is suitable for the production of various medical products due to its unique properties, such as smooth appearance, low toxicity, favorable mechanical properties (especially a low deformation effect) and high geometric resolution (Pajarito et al., 2019; Vicente et al., 2019). There are several published works on this topic (Lasprilla et al., 2012; Elmowafy et al., 2019; Singhvi et al., 2019), including our previous study dealing with PLA 3D printed safety masks during the COVID-19 pandemic (Vaňková et al., 2020) or other studies evaluating the use of PLA for 3D printing of tissue scaffolds (Zhang and King, 2020; Grigora et al., 2023). However, the 3D printed PLA objects can be easily colonized by various microorganisms due to cavities in their microstructure. Although disinfection of the 3D printed PLA objects with common chemicals is feasible (Vaňková et al., 2020), a self-disinfecting surface offers a low maintenance operation, high handling safety while omitting material degradation by chemicals or other conventional degradation techniques.

A functional antimicrobial layer added on the surface of PLA would represent a solution and further increase the usability of these products. One way to functionalize the surface of 3D printed PLA objects (Knetsch and Koole, 2011; Casalini et al., 2019; Elmowafy et al., 2019) is to coat them with a smooth metallic coating or metallic nanoparticles (NPs) with antimicrobial properties. Among metals, silver and especially its nanoparticles (AgNPs) have been often investigated for their possible medical use due to their excellent oligodynamic antimicrobial effect (Marambio-Jones and Hoek, 2010; Sotiriou and Pratsinis, 2010; Kalwar and Shan, 2018; Kumar et al., 2022). The oligodynamic effect (Prasher et al., 2018) occurs at low concentrations of heavy metals, where silver was identified as one of the most suitable for a variety of applications. AgNPs are capable of penetration into cells, and generate the reactive oxygen species and free radicals. For viruses (e.g., *Coronaviridae*), the ions can also bind to the spike glycoprotein, thus preventing its binding to host cells (Salleh et al., 2020). In the case of bacteria, AgNPs can penetrate the outer membrane and accumulate in the inner membrane, where the adhesion of NPs to the cell leads to destabilization and damage of the membrane, increasing its permeability, leading to leakage of cell contents and subsequent cell death (Bruna et al., 2021; Tripathi and Goshisht, 2022). There are a large number of studies describing the antimicrobial effect of AgNPs, e.g., when coated on polyurethane film (Prabhakar et al., 2011), stainless steel as implant materials (Furqon et al., 2021), paper (Srikhao et al., 2022), *Eichhornia crassipes* fiber (Palai et al., 2022), or silk surgical sutures (Syukri et al., 2020). However, despite the strong antimicrobial effects, AgNPs may have a cytotoxic effect against mammalian cells mainly by disrupting the membranes, affecting ATP production and DNA replication, altering gene expression, and oxidizing biological compartments in cells due to the mentioned production of reactive oxygen species (Fahmy et al., 2019). This effect occurs, especially when AgNPs are detached from the surface (Singh and Ramarao, 2012). To prevent it, the layer of AgNPs can be covered with a very thin protective film of various kinds (Kylián et al., 2017; Kratochvíl et al., 2018a). One of the precursors suitable for the preparation of such an overcoating is hexamethyldisiloxane (HMDSO), whose main advantage is that the physicochemical properties of the resulting film can be easily tuned over a wide range from hydrophobic to hydrophilic films (Kuzminova et al., 2014). This protective film can improve adhesion of the layer, while allowing the diffusion of Ag^+^ ions, but preventing direct contact between the AgNPs and the surrounding environment. The resulting coating should possess antimicrobial properties and, due to better fixation on the substrate, also low toxicity to human cells (Kratochvíl et al., 2018a).

The preparation of various antimicrobial materials based on coating the substrate with a layer of AgNPs or their combination with other overcoatings has been described in a number of studies. An example could be the coating of papers with chitosan/agar-AgNPs (Balalakshmi et al., 2020), or polydopamine/chitosan/AgNPs coating of urinary catheter and titanium surfaces (Wang et al., 2020). Furthermore, similar studies denoting specific applications were published for antibacterial fabrics (Allehyani et al., 2022; Cheng et al., 2022) or paper for food packaging materials (He et al., 2021). In agriculture or food industry, the coating with hydroxypropyl methylcellulose with AgNPs (Vieira et al., 2020), polyvinylpyrrolidone based glycerosomes with AgNPs (Saravanakumar et al., 2020), and carboxymethyl cellulose and guar gum-based AgNPs (Hmmam et al., 2021) extended the shelf life of papaya, bell pepper, and mango, respectively. Other potential applications in medicine have been reported for the antibacterial surfaces of titanium implants (Thukkaram et al., 2020; Wei et al., 2022), urinary catheter (Yassin et al., 2019), or for the treatment of onychomycosis (Dantas et al., 2021). In addition, the antiviral activity of polyethyleneimine with AgNPs coated on fabric from a surgical mask, hygienic mask, and combined polypropylene-glass fibers from a HEPA H13 filter was also reported (Baselga et al., 2022).

The desired smooth metallic and metallic NP coatings can be prepared by various methods. Among of all possible coating methods, the vacuum method based on a magnetron sputtering and subsequent gas aggregation in the case of NP deposition was found to be more suitable than classical chemical methods, yielding layers of better purity due to the absence of solvents and stabilizing agents (Wegner et al., 2006).

Although this issue has been studied already (Kratochvíl et al., 2018a; Kumar et al., 2022), the actual ambient conditions for medical application of AgNPs have not been determined. For instance, the above mentioned protective masks (Vaňková et al., 2020) are surrounded by a dry environment from the outside and a rather humid or wet one from the inside (close to the body). While for applications as biological tissue scaffolds, the surrounding environment is wet, saline-like, and body temperature. Although most studies have focused on Ag, antimicrobial properties are also possessed by copper (Vincent et al., 2016), which is cheaper and naturally occurs in trace amounts in the human body, which is not the case with Ag (Liu et al., 2019). To our knowledge, this is the first study directly comparing the antimicrobial effects of smooth Ag or other metallic coatings and AgNPs and monitoring the potential antiviral activities of such materials at the same time. Since proper efficiency of metallic coatings requires wetting and the large variation in humidity and/or wetting that can be expected in a real-life environment, we tested both dry (containing only atmospheric moisture) and wet conditions.

The aim of this study is to address the above-mentioned gaps in knowledge regarding the potential bio-applications of Ag, AgNP, and Cu coatings, such as a direct comparison of their biological properties (cytotoxicity, antibacterial, and antiviral action) in one study and consideration of the actual ambient conditions under which they can be practically used. Therefore, we compared the cytotoxic, antibacterial, and antiviral effects of AgNP layers (with and without a plasma-polymerized hexamethyldisiloxane (ppHMDSO) protective film) with Ag or Cu metallic coatings. We used three ambient conditions that mimic medical use (dry and wet conditions and conditions that simulate the human body). Coated PLA surface morphology was visualized using scanning electron microscopy (SEM). Human lung epithelial cells A549 were used to test the cytotoxicity of smooth metallic and metallic NP coatings. The bacterium *Pseudomonas aeruginosa* PAO1 and human rhinovirus species A, both representatives of respiratory pathogens, were used to test the efficiency of the coatings.

## 2. Materials and methods

### 2.1. Preparation of PLA carriers using 3D printing

Polylactic acid (PLA) was purchased in the form of filament for fused deposition modeling 3D printing from Verbatim GmbH, Germany. Square carriers (1 × 1 cm, 0.2 cm high) were prepared using a 3D printer (Prusa i3 MK3, Czech Republic). The printing template was designed with Autocad Inventor. The printing parameters were used as for the standard configuration of the printer for 0.2 mm layer height (extrusion temperature = 215°C, platform temperature = 60°C, filament flow = 100%).

### 2.2. Preparation of metallic coatings on PLA substrates

#### 2.2.1. Metallic coatings on PLA substrates

**Figure 1:**
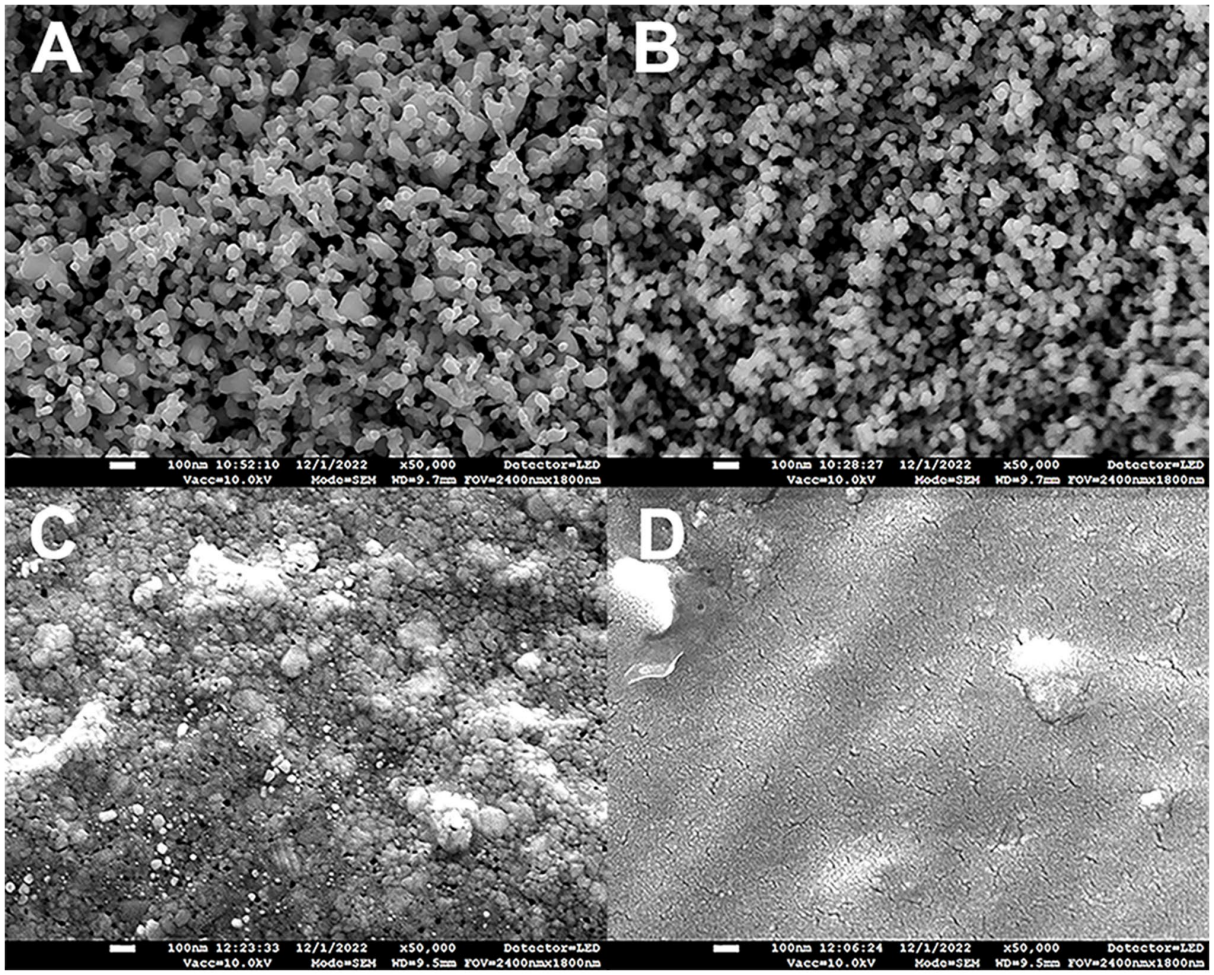
Scanning electron microscopy images of metallic coatings deposited on 3D printed polylactic acid (PLA) substrates **(A)** layer of silver nanoparticles (AgNPs), **(B)** layer of AgNPs overcoated by 16 nm of plasma-polymerized hexamethyldisiloxane (ppHMDSO), **(C)** silver coating (25 nm), **(D)** copper coating (25 nm). Scale bars 100 nm.

The metallic coatings were prepared in a high vacuum stainless steel chamber pumped by a turbomolecular (Pfeiffer THM 261P) and scroll (Edwards XDS10) pumps to the base pressure better than 1 × 10^−4^ Pa. Two different types of metallic coatings were prepared. First, Ag or Cu thin films were prepared by DC magnetron sputtering using 76 mm in diameter planar magnetron equipped with Ag (Safina a.s. 99.99% purity) or Cu (Kurt J. Lesker, 99.99% purity) target 3 mm in thickness. The magnetron was powered by a DC power supply (Advanced Energy MDX500) operated at a constant current mode. The current was fixed to 200 mA. Ar (99.996%, Linde Gas) was used as a working gas with a flow rate of 2 sccm, and the working pressure was adjusted to 4.4 Pa. Deposition time was set to 10 s and 15 s for Ag and Cu, respectively, to obtain coatings with the thickness of 25 nm. The second type of metallic coating was a multilayer of AgNPs (approximately 25 nm in size, Figure 1A). The NPs were produced in the so-called gas aggregation source (GAS) of nanoparticles. The method and experimental conditions were described in detail by Ahadi et al. (2022). Briefly, AgNPs were produced in a GAS equipped with a 76 mm in diameter planar magnetron with the silver target. Ar flow rate was set to 9 sccm with a corresponding pressure of 50 Pa in the aggregation chamber. The magnetron was powered by a DC power supply (Advanced Energy MDX500) at a constant current of 200 mA. The NPs were deposited on the PLA substrates in the main vacuum deposition chamber at a pressure lower than 0.1 Pa. The deposition time of AgNPs was set to 20 s which ensured the formation of a multilayer of the NPs.

#### 2.2.2. Deposition of ppHMDSO

Plasma polymerization (plasma enhanced chemical vapor deposition) was employed for the deposition of a ppHMDSO overcoating layer on AgNPs. The plasma polymerization took place in a tubular glass vacuum chamber 100 mm in diameter with external electrodes. The reactor was pumped by diffusion and rotary pumps to the base pressure better than 5 × 10^−3^ Pa. The capacitively coupled discharge was powered by a radio frequency (13.56 MHz) power supply (Advance Energy, Cesar) connected via a matching unit (MFJ-962D). A mixture of Ar and HMDSO (Ar/HMDSO = 6/1, total flow rate 3.5 sccm and total pressure of 3.5 Pa) was used as a working gas. The RF power was set to 60 W and the deposition time was chosen as 30 s to receive ppHMDSO film with a maximum thickness of 16 nm.

#### 2.2.3. Physical–chemical characterization of coatings

The chemical composition was investigated by means of X-ray photoelectron spectroscopy (XPS). The XPS spectrometer consists of X-ray source (Specs XR-50) with Al anode and hemispherical electron analyzer (Specs Phoibos 100). The X-ray source was operated at 12 kV and 200 W. The survey spectra recorded with the pass energy of 40 eV and resolution of 0.5 eV were used for the estimation of the chemical composition of resulting films. Data was processed using the CasaXPS software. The morphology of the resulting coating on the PLA substrates was visualized using scanning electron microscopy (SEM JEOL JSM-7900F). All images were taken at an electron beam voltage of 10 kV to minimize surface charging.

### 2.3. Cytotoxicity of metallic coatings determination

Human lung epithelial cells A549 (DSMZ ACC107) were cultured in DMEM (Dulbecco’s Modified Eagle Medium) with 10% FBS (fetal bovine serum) and Pen/Strep (Penicillin Streptomycin). For sterilization before experiments, all the PLA and coated PLA carriers (a square shape of 1 cm^2^) were immersed in ethanol for 20 min, removed and let dry (PLA carriers without any coating were used as control samples). Sterile PLA carriers were placed in a 24-well cell culture plate (round wells of 1.86 cm^2^) and about 1.5 × 10^5^ cells (absolute number of cells per well) were inoculated onto the carriers (covering both the carrier and its surrounding), wells were filled up to 500 μL with culture medium (phenol red-free). Additionally, cells seeded into a well without PLA carrier and medium without seeded cells were used as controls. Plates were incubated at 37°C in a CO_2_ incubator for 48 h. Subsequently, PLA carriers covered with cells were carefully transferred in a neighboring well and supplemented with 500 μL culture medium. In this way, cells grown on PLA carriers (“on carrier”) and those covering the surrounding well surface (“around carrier”) could be analyzed separately. MTT (3-(4,5-dimethylthiazol-2-yl)-2,5-diphenyl-2H-tetrazolium bromide, 60 μL, 12 mM) was added to each well. Plates were incubated at 37°C in a CO_2_ incubator for 2 h. Afterwards, cells were lysed by adding 10% SDS (sodium dodecyl sulfate, 500 μL) + HCl (hydrochloric acid, 0.01 N) and incubated overnight to achieve complete dissolving of the formed dye. Absorbance at 570 nm was measured using a PerkinElmer Enspire plate reader. Data were normalized for the equal area of cell growth, background (medium only control) was subtracted, and plotted as % of viability detected in cells grown without PLA carriers. All data were generated in three biological replicates and statistically analyzed using the Shapiro–Wilk test (to assess the data distribution). A value of *p* > 0.05 (in most cases very close to 1) indicated that the data follow a normal distribution. A *t*-test (two-sided test comparing coated carriers and uncoated control) was therefore employed to determine the significance of differences. Results displaying *p*-values < 0.05 were considered significant and depicted as average ± standard error of the mean.

### 2.4. Antibacterial activity of metallic coatings determination

The Gram-negative bacterium *P. aeruginosa* PAO1 was used as a representative of lung-infecting respiratory pathogens for antibacterial efficiency tests. For sterilization before experiments, all the PLA and coated PLA carriers (a square shape of 1 cm^2^) were immersed in ethanol for 20 min, removed and let dry (PLA carriers without any coating were used as control samples). Sterile PLA carriers were contaminated with 10 μL of bacterial suspension [approximately 1 × 10^7^ colony forming units (CFU) per mL] applied to the surface in 1 μL droplets and let dry under laminar flow to fix the bacteria on the surface. Further approach was different for each condition used. Inoculated carriers were: (1) immediately processed for further evaluation (simulating contamination in dry conditions), (2) covered with a saline (100 μL) for 4 h at room temperature without shaking and then processed for further evaluation (simulating contamination in wet conditions) or (3) cultivated in saline (1 mL) for 4 h at 37°C and 100 rpm and then processed for further evaluation (simulating environment inside the human body). To determine the bactericidal effect of the metallic coatings on PLA used under each condition, bacterial cells were collected in saline (1 mL) by vortexing the carriers. The obtained suspensions were decimally diluted, inoculated onto Luria-Bertani agar plates (LB; Oxoid, Czech Republic) and incubated at 37°C overnight. All data were generated in triplicate in three biological replicates and statistically analyzed using the Shapiro–Wilk test (to assess the data distribution). A value of *p* >0.05 in most cases very close to (1) indicated that the data follow a normal distribution. A *t*-test (two-sided test comparing coated carriers and uncoated control) was therefore employed to determine the significance of differences. Results displaying *p*-values < 0.05 were considered significant and depicted as average ± standard error of the mean.

### 2.5. Antiviral activity of metallic coatings determination

Human rhinovirus species A/type 2 (ATCC VR-482) was propagated in Hela Ohio cells (both a kind gift of Heinrich Kowalski) in DMEM with 10% FBS and Pen/Strep. For sterilization before experiments, all the PLA and coated PLA carriers (a square shape of 1 cm^2^) were immersed in ethanol for 20 min, removed and let dry (PLA carriers without any coating were used as control samples). Sterile PLA carriers were contaminated with 30 μL of virus suspension in a culture medium displaying a median tissue culture infectious dose (TCID) of 10^6^ infectious units (IU)/mL, applied in 6 droplets, each containing 5 μL solution. Further approach was as described above for antibacterial efficacy tests. Condition simulating the human body was not included, as virus particle survival requires host cells and independent long-term cultivation would therefore not be relevant. Contaminated carriers were let to dry (dry conditions) or were covered with 200 μL PBS (wet conditions) and incubated for 1 h. Subsequently, residual virus was recovered from the carrier surface directly (wet conditions) or using 200 μL PBS (dry conditions), the carrier was washed thoroughly: the virus-containing solution was repeatedly re-distributed across the entire surface. The recovered suspension was used directly for the infection of permissive cells to determine the infectious titer by a TCID50 (tissue culture infectious dose 50) assay using a 1:10 serial dilution in 96-well plates. Hela Ohio cells were incubated at 37°C in a CO_2_ incubator for 5 days, and virus-induced cytopathic effect was quantified using the Crystal Violet assay, as described previously (Lee et al., 2015). Calculation of the respective infectious titers expressed in IU/mL was performed using the Spearman-Kärber method (Kärber, 1931). Detection limit of the assay based on sample sizes of 30 μL is 84 IU. In addition, recovered genome copies were determined by quantitative real-time PCR (qRT-PCR), as described previously (Deffernez et al., 2004). All data were generated in three biological replicates and statistically analyzed using the Shapiro–Wilk test (to assess the data distribution). A value of *p* > 0.05 (in most cases very close to 1) indicated that the data follow a normal distribution. A *t*-test (two-sided test comparing coated carriers and uncoated control) was therefore employed to determine the significance of differences. Results displaying *p*-values < 0.05 were considered significant and depicted as average ± standard error of the mean.

## 3. Results

### 3.1. Morphology of metallic coatings on PLA substrates

Visualization of the samples by SEM (Figure 1) revealed that AgNP coatings on PLA substrates exhibit the typical highly porous structure of partially sintered AgNPs with the size approximately 25 nm (Figure 1A). As expected, the ppHMDSO film on the AgNP coatings did not significantly affect the primary structure of the coating (Figure 1B). However, the chemical composition of the surface, in this case, is primarily determined by the composition of ppHMDSO. Analysis of survey spectra from XPS indicated a presence of 66 at% C, 18 at% O and 16 at% Si. The deposition of a thin layer of Ag resulted in the formation of a film with a granular structure (Figure 1C). On the other hand, the sputtered Cu forms a compact, smooth film on the PLA surface (Figure 1D).

### 3.2. Cytotoxic effect of metallic coatings on human lung epithelial cells grown directly on carrier or in vicinity of carrier

**Figure 2:**
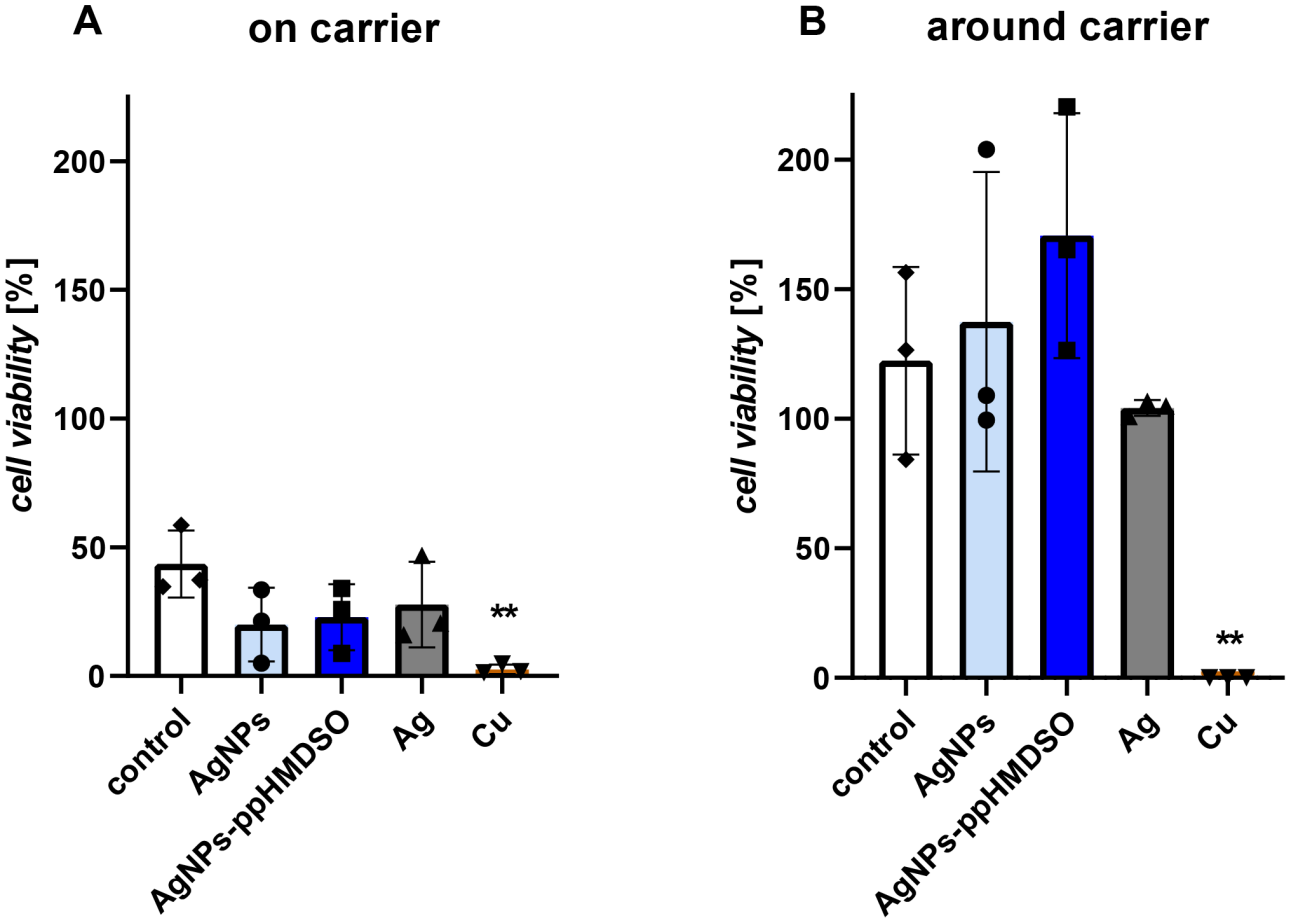
Cytotoxicity of metallic coatings on 3D printed polylactic acid (PLA) substrates on A549 cells seeded directly on the coated carrier **(A)** or around the coated carrier **(B)**. Control–uncoated PLA carrier, AgNPs–layer of silver nanoparticles, AgNPs-ppHMDSO–layer of silver nanoparticles overcoated by plasma-polymerized hexamethyldisiloxane (ppHMDSO), Ag–silver coating, Cu–copper coating. Cells were grown for 48 h and their viability was determined using an MTT (3-(4,5-dimethylthiazol-2-yl)-2,5-diphenyl-2H-tetrazolium bromide) assay. Results are depicted in % of the viability of cells growing on the same area inside a culture plate, three biological replicates ± standard error of the mean.

To determine the overall cytotoxicity of the studied metallic coatings on the human lung epithelial cell line A549, the cells were exposed to direct (cells grown on the carriers) and indirect (cells grown in the surrounding of the carriers) effects of AgNPs, AgNPs-ppHMDSO, Ag and Cu layers (Figure 2). As expected, only about 50% of cells grown standardly in culture wells were able to attach and grow directly on the uncoated carriers (Figure 2A). The Ag-based coated carriers (AgNPs, AgNPs-ppHMDSO, and Ag) supported growth less (approximately 25–30% of standardly grown cells) than the uncoated control, and even no cell growth was observed for the Cu-coated carriers. Cells that attached and grew around the carriers (Figure 2B) showed a much better profile in terms of metabolic activity, except for the Cu coating, which, similarly to cells attached directly on the carriers, completely abolished the metabolic activity, indicating a complete growth inhibition. Thus, Cu was completely blocking cell metabolism in both measurements, likely due to cytotoxicity. All the remaining metallic coatings (AgNPs, AgNPs-ppHMDSO and Ag) can be considered non-cytotoxic to the cells around them, since the metabolic activity of cells growing in their surroundings reaches at least 100% in all cases.

### 3.3. Antibacterial effect of metallic coatings on *Pseudomonas aeruginosa*

**Figure 3:**
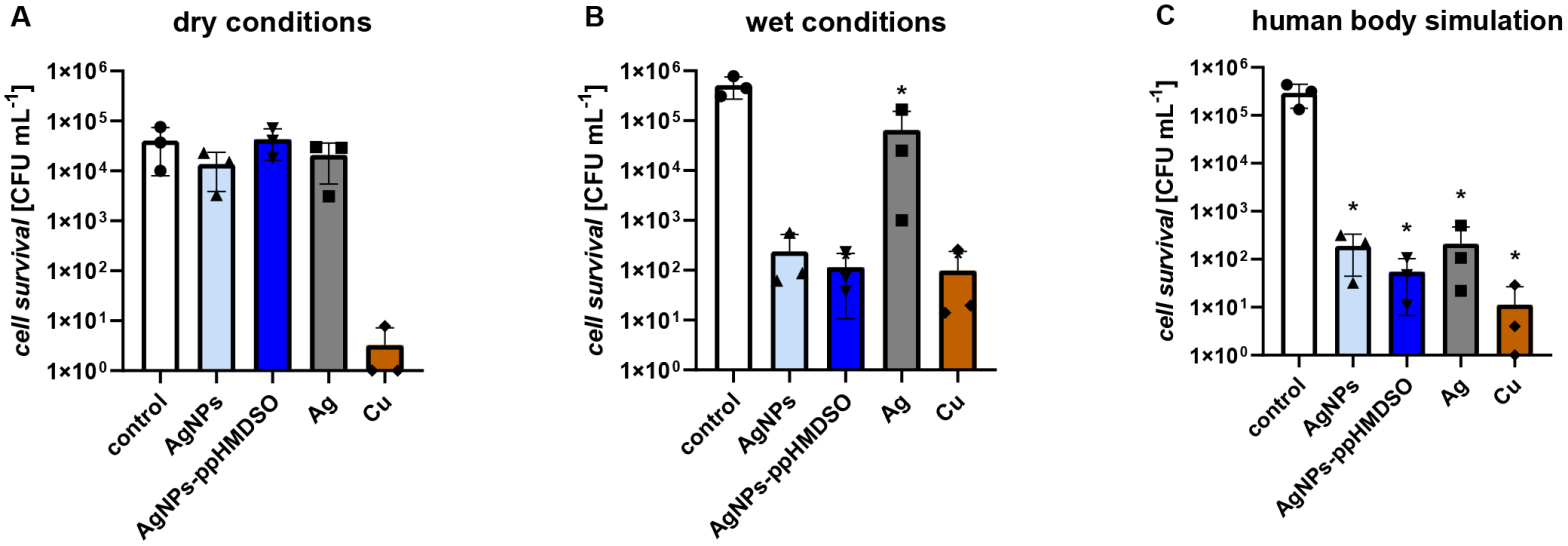
Antibacterial effect of metallic coatings of 3D printed polylactic acid (PLA) substrates against *Pseudomonas aeruginosa* PAO1. Control–uncoated PLA carrier, AgNPs–layer of silver nanoparticles, AgNPs-ppHMDSO–layer of silver nanoparticles overcoated with plasma-polymerized hexamethyldisiloxane (ppHMDSO), Ag–silver coating, Cu–copper coating. Bacterial inoculum (approximately 1 × 10^8^ CFU/mL) droplets were applied directly on the carriers and let dry **(A)** dry conditions; or let dry and then completely covered with 200 μL of saline for 4 h at room temperature without shaking **(B)** wet conditions; or let dry and then cultured in saline for 4 h at 37°C and 100 rpm **(C)** human body simulation. After the respective times, the bacterial cells were collected and colony forming units per milliliter (CFU/mL) were determined. Results are depicted in CFU/mL ± standard error of the mean.

To evaluate the antibacterial activity of the studied metallic coatings (AgNPs, AgNPs-ppHMDSO, Ag and Cu; Figure 3), *P. aeruginosa* PAO1 was selected as a model pathogenic bacterium infecting the lungs. The cell survival in the presence of metallic coatings on PLA substrates was studied under three different conditions–dry, wet and simulating the human body. As expected, cell survival in dried control samples (Figure 3A) was lower by one order of magnitude in CFU/mL than in wet conditions (Figure 3B) and saline-cultured samples (Figure 3C). However, none of the Ag-based coatings (AgNPs, AgNPs-HMDSO, and Ag) showed significant effectiveness in dry conditions (*p* < 0.05; Figure 3A), only the Cu layer almost completely inhibited cell growth. On the contrary, approximately 4 orders of magnitude reduction in CFU/mL was achieved in the case of the layer of AgNPs-ppHMDSO both in wet conditions or when cultured in saline simulating the human body environment (Figures 3B,C). A comparable effect was observed for the Cu layer, and only a slightly less effective one for the layer of AgNPs under these conditions. Interestingly, the Ag layer seemed ineffective in wet conditions (cell survival reached almost 1 × 10^5^ CFU/mL), but high bactericidal efficiency was observed in the case of the human body simulation (decrease by 3.5 orders of magnitude in CFU/mL).

### 3.4. Antiviral effect of metallic coatings toward human rhinovirus

**Figure 4:**
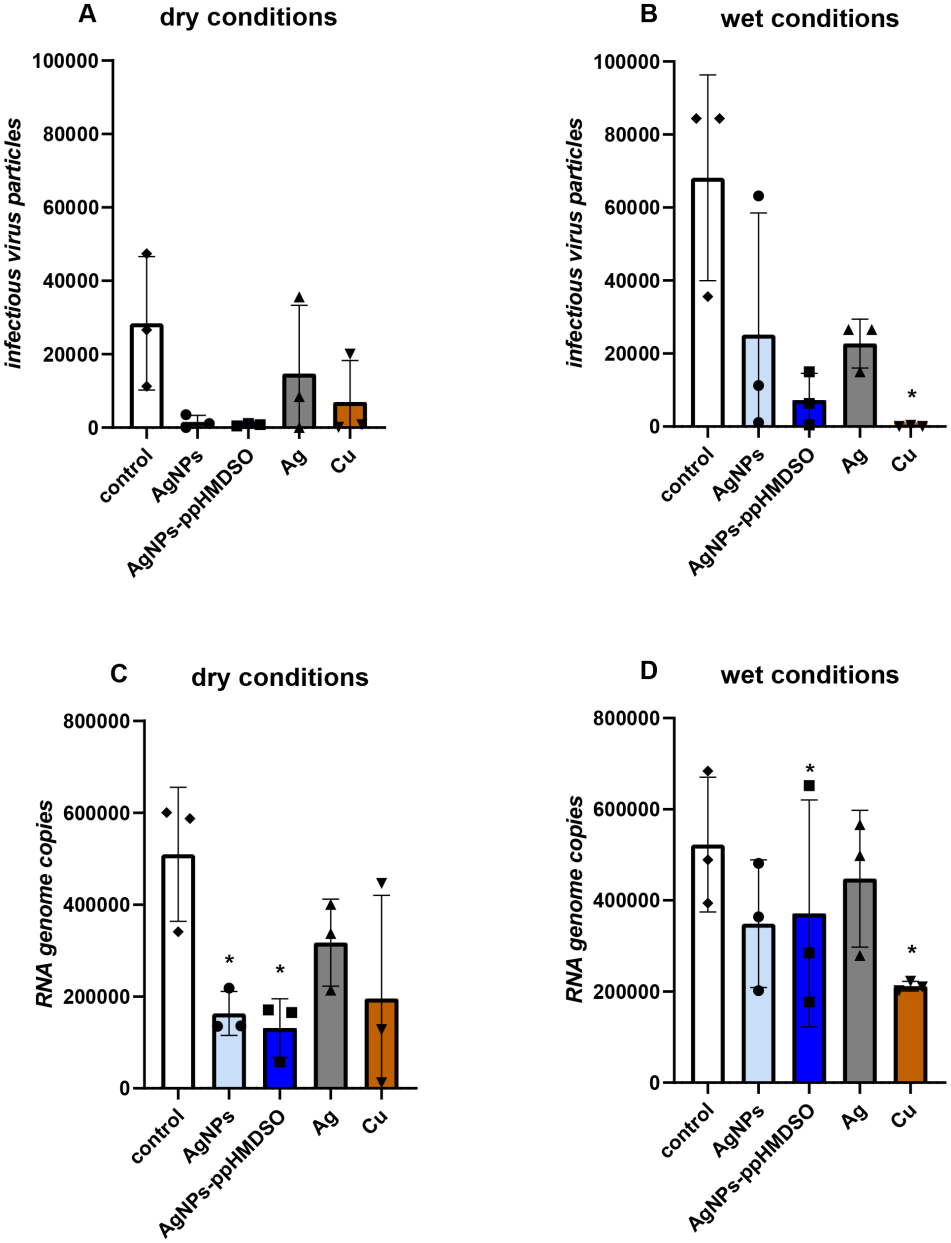
Antiviral effect of metallic coatings of 3D printed polylactic acid (PLA) substrates on human rhinovirus. Control - uncoated PLA carrier, AgNPs–layer of silver nanoparticles, AgNPs-ppHMDSO–layer of silver nanoparticles overcoated by plasma-polymerized hexamethyldisiloxane (ppHMDSO), Ag–silver coating, Cu–copper coating. Virus inoculum (10^6^ IU/mL) droplets were applied directly on the carriers and let dry **(A,C)** dry conditions; or covered with 200 μL of PBS **(B,D)** wet condition. After 1 h, residual virus particles were collected and their infectious titer **(A,B)** and genome copies **(C,D)** were determined. Results are depicted as the number of infectious virus particles or RNA genome copies ± standard error of the mean.

To address the antiviral activity of the studied metallic coatings (AgNPs, AgNPs-ppHMDSO, Ag and Cu; Figure 4), human rhinovirus type A/type 2 (ATCC VR-482) was used as a representative of the causative agents of respiratory diseases. As expected, the number of infectious virus particles in control samples was dramatically lower in the case of dry conditions (Figure 4A) than in wet conditions (Figure 4B). Despite this, the reduction of infectivity of the virus on different metallic coatings of PLA substrates under both conditions was very similar. The most effective antiviral coatings were AgNPs-ppHMDSO (complete inhibition in dry conditions and almost complete inhibition in wet conditions) and Cu (complete inhibition in wet conditions), while Ag layer exhibited the lowest efficiency. RNA genome copies, reflecting virus particle integrity, were slightly less reduced than infectious units under both conditions (Figures 4C,D). The largest decrease was observed in the case of dry conditions for AgNPs and AgNPs-ppHMDSO, and in wet conditions Cu achieved the highest efficiency. Ag layer was again the least effective in both cases. Virus titers fluctuated between individual biological replicates, which is an inherent property of complex biological experiments, resulting in only a limited number of statistically significant results.

## 4. Discussion

The study of NPs, their properties and use as active surfaces on various materials for application in medicine and other fields has been a highly attractive research topic in the last decades (Stark et al., 2015; Anselmo and Mitragotri, 2016; Anu Mary Ealia and Saravanakumar, 2017; Casalini et al., 2019; Elmowafy et al., 2019; Sánchez-López et al., 2020). Nevertheless, to our best knowledge, this is the first study that directly compares the biological effect of smooth Cu and Ag coatings to layers of AgNPs with and without a protective polymer (ppHMDSO) coating under different ambient conditions (dry and wet conditions and conditions simulating human body environment).

In general, the toxicity of AgNPs is strongly related to the particle size (ranging between 1 and 100 nm). Due to their extensive surface area, AgNPs are able to release a large amount of Ag^+^ ions, affecting the cells in their surroundings (Yin et al., 2020) Moreover, NPs of up to 10 nm in diameter can enter cells, increase the permeability of cell membranes, cause intracellular oxidative stress and even interrupt the replication of DNA (Marambio-Jones and Hoek, 2010; Kalwar and Shan, 2018; Yin et al., 2020; Kumar et al., 2022). The effects described are completely independent of cell type, applicable to human cells as well as bacteria (Sotiriou and Pratsinis, 2010; Quinteros et al., 2016; Kalwar and Shan, 2018). The mechanism of action of AgNPs is the subject of other scientific studies. For example, bacteria *Staphylococcus aureus* and *Escherichia coli* were treated by AgNPs powder and visualized using field emission SEM which shows not only morphological changes of cell surface but also cell fragments formed through damage of cell membrane (Hwan et al., 2010). Ashmore et al. (2018) treated *E. coli* with AgNP displayed an irregularly shaped appearance with nanoparticles attached to the cell membrane and fewer damaged or lysed cells. Ag^+^ ions are also implicated in the inhibition of uptake of phosphorus, causing the release of mannitol, phosphate, and amino acids such as proline and glutamine outside the cell membrane through the production of reactive oxygen species. The search for the mechanism of action was therefore not the focus of our study, in which the main objective was to compare the antibacterial and antiviral activity of individual metallic coatings under different conditions.

The Ag layers adhesion to the polymeric substrates is in general poor, but it is improved by enhanced substrate roughness and successful direct deposition of Ag thin film on PLA by magnetron sputtering was already reported (Da Silva et al., 2023). The advantage of AgNPs compared to a smooth Ag layer is their enormous active surface. The layer of AgNPs (approx. size of 25 nm, Figure 1A) visualized by SEM appeared very porous with partially sintered NPs, which is typical for these highly pure AgNP films, previously described by Ahadi et al. (2022). However, such coatings have poor mechanical stability, even worse than smooth Ag layers, since the NPs are held together only by Van der Waals forces and can be easily wiped off the substrates. Therefore, we tried to improve this property by coating the AgNPs with a thin ppHMDSO film anchoring the NPs on the substrates without significant changes in the coating structure. This layer can improve the anchoring of AgNPs on the PLA surface, as the nanocomposites in general are known to exhibit enhanced hardness and improved mechanical properties (Jedrzejowski et al., 2004). Indeed, based on the SEM images (Figures 1A,B), the surface morphology of AgNPs and AgNPs-ppHMDSO is basically the same. The polymeric film, which forms the top layer of the entire structure, can also affect the wettability of the coating and the release of Ag^+^ ions, thus changing the antibacterial properties of this surface, as described in a review by Kratochvíl et al. (2018a). The optimal balance between biological effects and biocompatible properties can be adjusted by both the proper film thickness and the chemical composition. In our study, the surface chemical composition of ppHMDSO samples, investigated by the XPS analysis (66 at% C, 18 at% O and 16 at% Si), is close to the values reported by Keller et al. (2007). In the case of smooth Ag layer, granular growth was observed by SEM due to the low wettability of the PLA substrate. Such behavior was described in more detail in previous work of our colleagues Kratochvíl et al. (2015).

In cytotoxicity studies, the Ag-based coatings seemed to act as a very poor support for cell growth (“on carrier,” Figure 2A). Therefore, cells were more likely to attach around it, leading to a relatively high metabolic activity in the “around carrier” dataset (Figure 2B). According to Gliga et al. (2014), 25 nm AgNPs should not be cytotoxic to lung epithelial cells, which is in agreement with our results using AgNPs coating (Figures 2A,B). A cytotoxicity study of Ag-based dressings on human fibroblast showed a similar cell behavior when in contact with material releasing Ag^+^ ions (Burd et al., 2007). A detailed study of the toxicity of AgNPs covalently stabilized by a small peptide that includes visualization of THP-1-derived human macrophages exposed to AgNPs using transmission electron microscopy or confocal Raman microscopy was published by Haase et al. (2011). The layer of AgNPs used in our study showed fluctuating cytotoxicity, but overcoating with ppHMDSO reduced this effect.

As mentioned above, an additional effect of very small NPs (up to 10 nm) is the induction of oxidative stress inside cells and affecting of DNA replication (Sotiriou and Pratsinis, 2010; Kalwar and Shan, 2018). The AgNPs investigated in this study are relatively large (25 nm), therefore, their direct induction of oxidative stress is rather unlikely, and the main effect on *P. aeruginosa* cells is probably caused by the release of Ag^+^ ions (Sotiriou and Pratsinis, 2010; Knetsch and Koole, 2011). Accordingly, our results showed that the layer of AgNPs possessed a stronger antibacterial effect (Figure 3) than the smooth Ag layer, likely due to the larger surface area and thus increased release of Ag^+^ ions. In support of the above-described notion that in dry conditions, release of ions is reduced and air humidity (RH about 40%) is not sufficient for considerable antibacterial activity of Ag-based coatings, we observed a generally weaker antibacterial effect under dry conditions. The antibacterial effects were very similar under conditions simulating the human body and wet conditions, with the exception of the smooth Ag layer, which had a much stronger antibacterial effect in saline than in wet conditions. This could be due to increased Ag^+^ ions release facilitated by a higher processing temperature and the stirring of the sample in the saline.

Regarding the antiviral impact of metallic coatings, our tests showed that both rhinovirus infectivity and genome integrity were reduced by all analyzed active layers (Figure 4). These results point toward a complex damage inflicted on the virus particles, which is consistent with previous studies using AgNPs (Salleh et al., 2020), Ag and Cu compounds (Minoshima et al., 2016). As expected, the infectivity was decreased to a higher degree than genome integrity, which is likely to be reduced only when the viral particles are severely damaged. The layer of AgNPs-ppHMDSO coating maintained similar antiviral effects to the layer of AgNPs. Its favorable cytotoxicity profile (Figure 2) and antibacterial activity (Figure 3) make the AgNPs-ppHMDSO combination very promising for medical applications. A similar conclusion emanated from a study of antibacterial activity of CuNPs on polyether-ether-ketone substrates overcoated with a polytetrafluorethylene using *Escherichia coli* ATCC 25922, complemented by a cytotoxicity study on human osteosarcoma cells MG63 (Kratochvíl et al., 2018b). They showed that by choosing appropriate conditions (mainly the amount of NPs and the thickness of the polymeric film), it is possible to create a material with simultaneously antibacterial and biocompatible properties.

Compared to the Ag-based layers (AgNPs, AgNPs-ppHMDSO, Ag) tested in our study, the smooth Cu layer was generally highly antimicrobial effective, but at the same time highly cytotoxic. Cu layer without any protective coating used in our study was clearly highly cytotoxic in direct (“on carrier“) and indirect (“around carrier“) contact (Figure 2), which is consistent with other papers concerning the cytotoxicity of Cu and its alloys on different cell types (Craig and Hanks, 1990; Wigle, 1992; Suska et al., 2005; Čolić et al., 2010). The Cu layer displays a high level of toxicity, not only to eukaryotic, but also bacterial cells or virus particles. The copper coating appears to be, according to our experiments, the most effective antibacterial agent that inhibits bacterial cells even in dry conditions (Figure 3). Moreover, despite fluctuations in the data, a clear trend toward a robust antiviral effect of the Cu layer in both dry and wet conditions is visible (Figure 4). This is consistent with several studies on the antiviral impact of copper, such as Noyce et al. (2007), who reported rapid inactivation of influenza A virus on the copper surface or the review reporting the effect of Cu and its alloys on SARS-CoV-2 virus (Govind et al., 2021). The Cu coating in our study was selected as a cheaper alternative to Ag layers, but its profound cytotoxicity (Figure 2) prevents applications in contact with the human body.

The obtained results in summary indicate that the AgNPs-ppHMDSO coatings represent an ideal surface for various types of implants, scaffolds and other intracorporal applications. On the other hand, the Cu layer could be practical for coating medical instruments in which cytotoxicity can be tolerated, as it is an efficient antibacterial and antiviral agent.

## 5. Conclusion

This study directly compares the biological properties of four metallic coatings (AgNP coating, AgNPs overcoated with a protective ppHMDSO film, Ag and Cu metallic coatings) and identifies the most suitable ones for medical applications. The obtained results show that regardless of the ambient environment, Cu is the most efficient antimicrobial agent, but highly cytotoxic at the same time. In summary, these properties make it suitable for use outside of the human body, efficient even in dry conditions. For all Ag-based coatings, the antimicrobial properties are greatly enhanced in the presence of liquid (wet conditions and environment simulating the human body). Moreover, the AgNPs appear to be more effective against bacterial cells and viral particles than the smooth Ag layer. The antibacterial and antiviral activity of AgNPs covered by the ppHMDSO film is generally comparable to or slightly higher than pure AgNPs. Together with the fact that the ppHMDSO coating minimizes the cytotoxicity of the AgNPs, these layers are promising for applications in close contact with or even inside the human body. Overall, the layer of AgNPs-ppHMDSO is non-toxic to lung epithelial tissue cells in its vicinity and effective against *P. aeruginosa* and rhinovirus. Therefore, this coating represents an excellent choice for a simple modification of 3D printed instruments applicable, for example, as various scaffolds inserted into the human body.

## Data availability statement

The raw data supporting the conclusions of this article will be made available by the authors, without undue reservation.

## Author contributions

AM, EV, and KO were responsible for conceptualization and design of the study. EV and KO were responsible for the project administration. AM, PF, KO, and TK were responsible for the investigation and methodology. KO performed the statistical analysis. AM wrote the first draft of the manuscript. EV, KO, and JH wrote sections of the manuscript. EV, KO, JH, TL, and VS were responsible for funding and supervision. All authors contributed to the review and editing of the manuscript and approved the submitted version.

## Funding

This study was financially supported by the FWF/GACR grant FWF I 5293-B/GF21-39019L.

## Conflict of interest

The authors declare that the research was conducted in the absence of any commercial or financial relationships that could be construed as a potential conflict of interest.

## Publisher’s note

All claims expressed in this article are solely those of the authors and do not necessarily represent those of their affiliated organizations, or those of the publisher, the editors and the reviewers. Any product that may be evaluated in this article, or claim that may be made by its manufacturer, is not guaranteed or endorsed by the publisher.
